# Discovery of Novel Amino Acids (Analogues)-Substituted Thiophene[3,2-*d*]pyrimidine Derivatives as Potent HIV-1 Non-Nucleoside Reverse Transcriptase Inhibitors: Design, Synthesis, and Biological Evaluation

**DOI:** 10.3390/ijms25169028

**Published:** 2024-08-20

**Authors:** Zongji Zhuo, Zhao Wang, Lanlan Jing, Tao Zhang, Anchao Ge, Zhenzhen Zhou, Ying Liu, Xin Li, Erik De Clercq, Christophe Pannecouque, Peng Zhan, Xinyong Liu, Dongwei Kang

**Affiliations:** 1 Key Laboratory of Chemical Biology (Ministry of Education), Department of Medicinal Chemistry, School of Pharmaceutical Sciences, Cheeloo College of Medicine, Shandong University, 44 West Culture Road, Jinan 250012, China; 202236926@mail.sdu.edu.cn (Z.Z.); wangzhao@sdu.edu.cn (Z.W.); 202020834@mail.sdu.edu.cn (L.J.); 202221043@mail.sdu.edu.cn (T.Z.); 202316722@mail.sdu.edu.cn (A.G.); 202136924@mail.sdu.edu.cn (Z.Z.); 202336816@mail.sdu.edu.cn (Y.L.); 202336806@mail.sdu.edu.cn (X.L.); zhanpeng1982@sdu.edu.cn (P.Z.); 2 Laboratory of Virology and Chemotherapy, Rega Institute for Medical Research, K.U. Leuven, Herestraat 49 Postbus 1043 (09.A097), B-3000 Leuven, Belgium; erik.declercq@kuleuven.be (E.D.C.); christophe.pannecouque@kuleuven.be (C.P.); 3 China-Belgium Collaborative Research Center for Innovative Antiviral Drugs of Shandong Province, Shandong University, 44 West Culture Road, Jinan 250012, China

**Keywords:** HIV-1, NNRTIs, thiophene[3,2-*d*]pyrimidine, amino acids, drug design

## Abstract

Inspired by our previous work on the modification of diarylpyrimidine-typed non-nucleoside reverse transcriptase inhibitors (NNRTIs) and the reported crystallographic studies, a series of novel amino acids (analogues)-substituted thiophene[3,2-*d*]pyrimidine derivatives were designed and synthesized by targeting the solvent-exposed region of the NNRTI-binding pocket. The biological evaluation results showed that compound **5k** was the most active inhibitor, exhibiting moderate-to-excellent potency against HIV-1 wild-type (WT) and a panel of NNRTI-resistant strains, with EC_50_ values ranging from 0.042 μM to 7.530 μM. Of special note, **5k** exhibited the most potent activity against single-mutant strains (K103N and E138K), with EC_50_ values of 0.031 μM and 0.094 μM, being about 4.3-fold superior to EFV (EC_50_ = 0.132 μM) and 1.9-fold superior to NVP (EC_50_ = 0.181 μM), respectively. In addition, **5k** demonstrated lower cytotoxicity (CC_50_ = 27.9 μM) and higher selectivity index values. The HIV-1 reverse transcriptase (RT) inhibition assay was further performed to confirm their binding target. Moreover, preliminary structure–activity relationships (SARs) and molecular docking studies were also discussed in order to provide valuable insights for further structural optimizations. In summary, **5k** turned out to be a promising NNRTI lead compound for further investigations of treatments for HIV-1 infections.

## 1. Introduction

**Figure 1:**
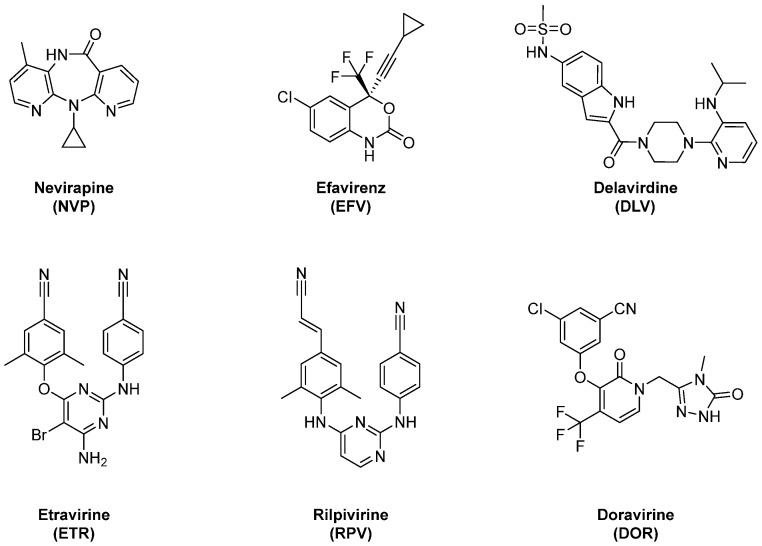
Chemical structures of six NNRTIs approved by the U.S. FDA.

Acquired immune deficiency syndrome (AIDS), caused by human immunodeficiency virus-1 (HIV-1), seriously threatens human health worldwide. According to the latest global epidemiological statistics released by the World Health Organization (WHO), there are currently approximately 39.0 million people living with HIV, including 1.3 million new HIV infections in 2022 [1]. In the replication cycle of HIV-1, reverse transcriptase (RT) is responsible for reverse transcription of single-stranded RNA into double-stranded DNA; this was a preferred target for drug design for its clear mechanism of action and abundant structural biology information [2,3]. Based on their mechanisms of action, the RT inhibitors can be divided into nucleoside RT inhibitors (NRTIs) and non-nucleoside RT inhibitors (NNRTIs) [4,5]. NNRTIs possess stronger antiviral potency, lower toxicity and high selectivity [6,7,8,9,10,11,12,13]. Up to now, six NNRTIs have been approved by the U.S. Food and Drug Administration (FDA), including the first-generation drugs nevirapine (NVP), delavirdine (DLV), and efavirenz (EFV), and the second-generation drugs etravirine (ETR), rilpivirine (RPV), and doravirine (DOR) (Figure 1) [10,14]. Among them, ETR and RPV are part of the diarylpyrimidine (DAPY) family and exhibit potent activity against clinically relevant NNRTIs-resistant strains [15]. However, the emergence of single mutant strains (such as K103N and Y181C) has severely limited the clinical use of the first-generation NNRTIs [15,16,17]. Although the second-generation NNRTIs have shown some improvement as to the activity of single mutant strains, the emergence of new single mutant (E138K) and double mutant (RES056) strains and serious adverse effects have limited their clinical application [15,16]. Therefore, new NNRTIs that offer a combination of improved potency against these mutants, a favorable profile of safety, and tolerability is urgently needed.

**Figure 2:**
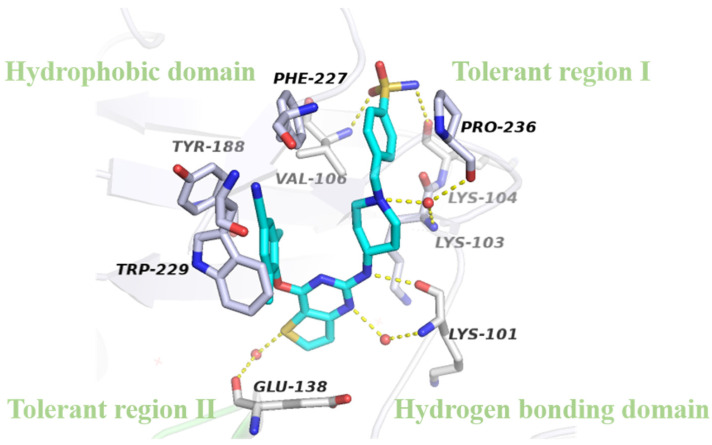
The detailed interactions with WT RT of **K-5a2** and the four-point pharmacophore mode (PDB code: 6C0J).

Our previous efforts have identified a series of new DAPY derivatives with a more potent antiviral activity against WT and mutant HIV-1 strains than that of the approved drug ETR [18]. In particular, **K-5a2** exhibited highly effective anti-HIV-1 activities and improved resistance profiles. The co-crystal structure of HIV-1 RT/**K-5a2** confirmed the “four-point pharmacophore model” of DAPY in NNIBP, including the hydrophobic domain, the hydrogen bonding domain, tolerant region I and tolerant region II [18]. Concretely, the interactions between **K-5a2** and RT have been delineated. As shown in Figure 2, the thiophene[3,2-*d*]pyrimidine central scaffold could form a hydrogen bond with K101 and E138 through a bridging water molecule, the left wing projected into the hydrophobic channel and contacted with Tyr188 and Trp229 by *π–π* interaction, the piperidine nitrogen develop water-mediated hydrogen bonds with K103 and P236, and the surface-positioned sulfonamide group of the right wing establish a double-hydrogen bond with the carbonyl oxygen of K104 and the backbone nitrogen of V106. Solvent-exposed regions provide opportunities for substantial modifications of current small-molecular drugs; these regions were often considered as potential binding sites for the optimization of a chemical series to acquire additional and specific protein–ligand interactions and to enhance affinity for a target protein [19].

**Figure 3:**
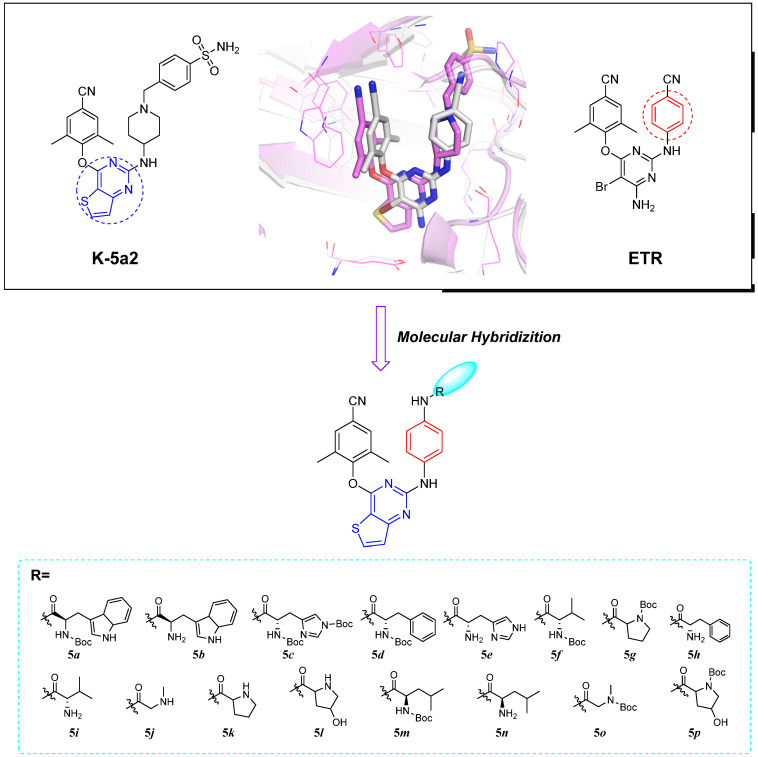
Design of the novel thiophene[3,2-*d*]pyrimidine derivatives.

In our previous work, the central thiophene[3,2-*d*]pyrimidine has been determined as a privileged scaffold for improving antiviral activity, and it can occupy the NNIBP more extensively and has generated stronger interactions with RT [19]. Thus, the dominant thiophene pyrimidine ring was retained. On the other hand, we discarded the piperidine-linked benzenesulfonamide scaffold of **K-5a2** that perturbs hERG and retained the benzene right wing of ETR based on the overlap between ETR/HIV-1 RT and **K-5a2**/HIV-1 RT. In addition, variety of amino acids and their analogues were introduced at the terminal of the right-wing benzene ring, with the aim of enhancing binding affinity and antiviral activity through improving hydrogen bond donor–acceptor interactions, while exploring the chemical space within the solvent-exposed regions and enriching the structure–activity relationship (SAR). In the present work, guided by the available crystallographic studies of the **K-5a2**/RT complex, a series of novel amino acids (analogues)-substituted thiophene[3,2-*d*]pyrimidine derivatives were rationally designed by targeting the solvent-exposed region of the NNIBP. And the design of the novel thiophene[3,2-*d*]pyrimidine derivatives was showed in Figure 3.

## 2. Results and Discussion

### 2.1. Chemistry

**Scheme 1:**
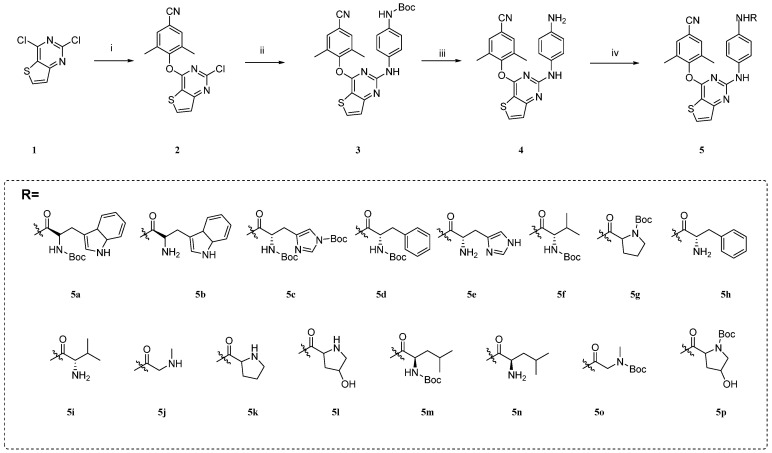
Synthesis of **5a**–**p**. Reagents and conditions: (i) DMF, K_2_CO_3_, r.t., 5 h; (ii) BINAP, Pd_2_(dba)_3_, Cs_2_CO_3_, 1,4-dioxane, 120 °C, N_2_, 14 h; (iii) TFA, DCM, r.t., 4 h; (iv) HATU, DIEA, CH_2_Cl_2_, 0 °C to r.t., 8–10 h.

The synthetic protocols for the newly designed compounds **5a–p** are outlined in Scheme 1. The 2,4-dichlorothieno[3,2-*d*]pyrimidine was selected as the starting material, which was reacted with 4-hydroxy-3,5-dimethylbenzonitrile through nucleophilic substitution to afford intermediate **2**. Then, intermediate **2** was reacted with tert-butyl (4-aminophenyl)carbamate in the presence of BINAP and Pd_2_(dba)_3_ to yield the intermediate **3** via Buchwald–Hartwig reaction. Then, **3** was reacted with trifluoroacetic acid (TFA) to generate **4**. Finally, the target compounds **5a–p** were obtained by amide condensation reaction of **4** with different amino acids in the presence of HATU and DIEA at 0 °C to room temperature (r.t.).

### 2.2. Anti-HIV Activity Evaluation

All the target compounds were first evaluated for their antiviral activity against HIV-1 wild-type (WT) strain (IIIB) and the double-mutant strain K103N+Y181C (RES056) in the MT-4 cell line, using the MTT method. The potent inhibitors were selected to assess their efficacy against other strains resistant to NNRTIs, including L100I, K103N, Y181C, Y188L, E138K and the double-mutant strain F227L+V106A. The approved drugs NVP, EFV and ETR were selected as reference drugs. The biological evaluation results of the test compounds, presented as EC_50_ (anti-HIV potency), CC_50_ (cytotoxicity) and SI (selectivity index, CC_50_/EC_50_ ratio), are summarized in Table 1 and Table 2.

As shown in Table 1, most compounds exhibited promising potency against WT HIV-1 strain, with EC_50_ values ranging from 0.042 μM to 0.940 μM. Compounds **5e, 5h, 5i, 5k, 5l** and **5n** yielded the most potent activity against WT HIV-1 strain with EC_50_ values in the range of 0.042–0.062 μM, which were not only much superior to that of NVP (EC_50_ = 0.236 μM), but also comparable to that of ETR (EC_50_ = 0.003 μM). Among them, **5k** (CC_50_ = 27.901 μM, SI = 513) exhibited much lower cytotoxicity and higher SI values against the WT HIV-1 strain. However, the antiviral activity of most compounds against the double mutant strain RES056 was relatively limited. According to the anti-HIV-1 IIIB results, preliminary structure–activity relationships (SARs) analysis was summarized as follows. The antiviral efficacy was diminished with R substituents featuring a Boc group, as observed in comparisons between **5b** and **5a**, **5e** and **5c**, **5h** and **5d**, **5i** and **5f**, **5k** and **5g**, **5n** and **5m**, **5j** and **5o**, and **5l** and **5p**. Furthermore, R substituents with a non-polar amino acid group demonstrated superior antiviral potency compared to their polar counterparts. Furthermore, the results of the selected compounds against a variety of NNRTIs-resistant strains (L100I, K103N, Y181C, Y188L, E138K and F227L + V106A) are summarized in Table 2. As for K103N, the most prevalent resistance-associated mutation to NVP and EFV, all the selected compounds displayed potent activities (EC_50_ = 0.031–0.131 μM) and were superior to that of NVP (EC_50_ = 4.091 μM). Particularly, compound **5k** (EC_50_ = 0.031 μM) provided the most potency, which was not only far superior to that of NVP but also 4.25-fold greater than that of EFV (EC_50_ = 0.132 μM). Moreover, **5k** also showed highly potent activity against E138K, with an EC_50_ value of 0.094 μM, a level superior to that of NVP (EC_50_ = 0.181 μM). As for Y188L strain, all the compounds exhibited prominent EC_50_ values, ranging from 2.11 μM to 6.907 μM, being more potent than that of NVP (EC_50_ = 9.868 μM). In the cases of L100I and Y181C, **5k** (EC_50_ = 0.601 μM, EC_50_ = 1.847 μM) also showed the best antiviral activity, being superior to that of NVP (EC_50_ = 1.741 μM, EC_50_ = 8.035 μM). As for F227L + V106A, **5e** (EC_50_ = 1.465 μM) exhibited a much more potent activity than that of NVP (EC_50_ = 7.499 μM).

### 2.3. Inhibition of HIV-1 RT

To validate the binding target of these novel compounds, some representative compounds were selected in order to evaluate their inhibitory effects on WT HIV-1 RT, with NVP, EFV and ETR as reference drugs. As depicted in Table 3, all tested compounds exhibited potent inhibitory activity against WT HIV-1 RT, with IC_50_ values in the range of 0.972–2.390 μM, levels comparable to that of NVP (IC_50_ = 0.735 μM). Therefore, the HIV-1 RT inhibition assay results demonstrated that these novel thiophene[3,2-*d*]pyrimidine derivatives have high binding affinity for HIV-1 RT and belong to classical NNRTIs.

### 2.4. Molecular Modeling Analysis

With the aim of better explaining the anti-HIV potency and to obtain further insights into the binding modes, the molecular modeling studies of the most active compound, **5k**, were carried out by utilizing the Maestro software (Maestro, Schrödinger, LLC, New York, NY, USA, 2019). Co-crystal structures of HIV-1 WT RT (PDB code: 6C0J), K103N RT (PDB code: 6C0K) and E138K RT (PDB code: 6C0L) were selected as the input structures for molecular docking calculations. The docking results were visualized with PyMOL.

**Figure 4:**
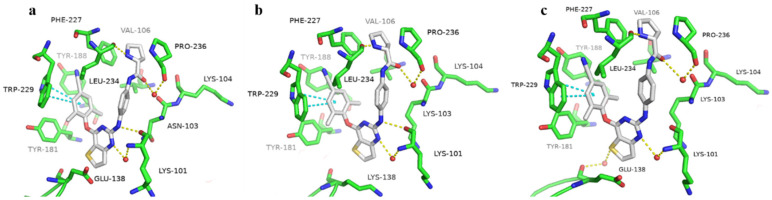
(**a**–**c**) Predicted binding modes of **5k** (sliver) with the HIV-1 K103N RT (PDB code: 6C0K), E138K RT (PDB code: 6C0L) and HIV-1 WT RT (PDB code: 6C0J), respectively.

As shown in Figure 4, **5k** still followed the classical horseshoe conformation of DAPY derivatives in the NNIBP. Specifically, the docking result can be summarized as follows: (1) The N atom of thienopyrimidine group remained to form a hydrogen bond with Lys101 through a bridging water molecule, and the linker N atom still established a hydrogen bond with Lys101 residue. (2) Hydrophobic contacts between the left wing and Trp229 still remained. (3) The right wing formed a rich hydrogen bonding interaction in the solvent-exposed region, which was more favorable to the inhibition of RT. We next predicted the binding mode of **5k** with single mutation K103N RT and E138K RT. Carbonyl oxygen atoms formed hydrogen bonding interactions with Pro236 via water bridges, and the N atom of Proline established hydrogen bond with Leu234, which may account for the better antiviral activity against these two single mutant strains. It was noteworthy that **5k** lost crucial water-mediated hydrogen bonds with Glu138 in K103N RT, resulting in a reduced potency compared to **K-5a2**.

## 3. Methods and Materials

### 3.1. Chemistry

Melting points were ascertained using a micro melting point apparatus (RY-1G, Tianjin Tian Guang Optical Instruments Company, Tianjin, China). Nuclear magnetic resonance (NMR) spectra, both ^1^H and ^13^C, were obtained using a Bruker AV-400 instrument (Bruker Corporation, Billerica, MA, USA) in DMSO-*d*_6_, with tetramethylsilane (TMS) as a reference. Signal multiplicities are denoted by s (singlet), d (doublet), t (triplet) and m (multiplet). Chemical shifts are presented in δ (parts per million) relative to TMS, with coupling constants specified in hertz (Hz). Mass spectrometry was performed on an Agilent AG1313A Standard LC Autosampler(Agilent Technologies Comppany, Santa Clara, CA, USA).

Routine monitoring of all reactions was performed via thin layer chromatography (TLC) on Merck’s Silica Gel GF254 plates, with visualization of spots achieved through iodine staining or UV light exposure at wavelengths of 254 and 356 nm. Flash column chromatography utilized columns filled with silica gel from Qingdao Haiyang Chemical Company (Qingdao, Shandong, China). Solvent purification and drying followed standard protocols, and reaction solution concentration was facilitated by rotary evaporation under vacuum conditions.

General Synthesis Procedure for intermediate **2**.

A reaction mixture of 2,4-dichlorothieno[3,2-*d*]pyrimidine and 4-hydroxy-3,5-dimethylbenzonitrile in 30 mL of N, N-Dimethylformamide (DMF) was stirred at room temperature for 5 h. After completion of the reaction (monitored by TLC), the precipitated white solid was collected by filtration, washed with ice water (200 mL), and recrystallized in DMF-H_2_O to provide the intermediate **2**.

4-((2-chlorothieno[3,2-*d*]pyrimidin-4-yl)oxy)-3,5-dimethylbenzonitrile (**2**) White solid, yield: 94%, mp: 258–260 °C. ^1^H NMR (400 MHz, DMSO-*d*_6_) δ 8.61 (d, *J* = 5.4 Hz, 1H, C6-thienopyrimidine-H), 7.78 (s, 2H, Ph-H), 7.70 (d, *J* = 5.4 Hz, 1H, C7-thienopyrimidine-H), 2.12 (s, 6H, CH_3_×2). ESI-MS: m/z 316.08 [M + H]^+^. C_15_H_10_ClN_3_OS (315.02)

General Synthesis Procedure for intermediate **4.**

A reaction mixture of Pd_2_(dba)_3_ (0.30 g, 0.32 mmol) and BINAP (0.2 g, 0.32 mmol) in 10 mL of dry 1,4-dioxane was stirred at room temperature for 15 min, and then tert-butyl (4-aminophenyl)carbamate (0.11 g, 0.53 mmol) and Cs_2_CO_3_ (0.31 g, 0.95 mmol) were added. Stirring was continued for an additional 10 min; then, intermediate **2** was added. The flask was evacuated and backfilled with nitrogen. The mixture was stirred at 120 °C for another 14 h (monitored by TLC). Then, the solvent was evaporated under reduced pressure, and the obtained residue was dissolved in 20 mL of ethyl acetate (EA). The organic phase was washed with saturated sodium chloride (35 mL), dried over anhydrous Na_2_SO_4_, filtered and then purified by flash column chromatography to give the intermediate **3**. Then intermediate **3** (0.10 g, 0.21 mmol) and TFA (1 mL, 7.5 mmol) were stirred at room temperature for 4 h in dry DCM (6 mL). Subsequently, the mixture was adjusted to a basic pH of 9 using sodium bicarbonate (NaHCO_3_) and then extracted with dichloromethane (DCM). The consolidated DCM extracts were subjected to flash column chromatography, resulting in the isolation of the crucial intermediate compound 4.

4-((2-((4-aminophenyl)amino)thieno[3,2-d]pyrimidin-4-yl)oxy)-3,5-dimethylbenzonitrile (**4**) White solid, yield: 65%, mp: 176–178 °C. ^1^H NMR (400 MHz, DMSO-*d*_6_) δ 9.66 (s, 1H, NH), 8.37 (d, *J* = 5.4 Hz, 1H, C6-thienopyrimidine-H), 7.79 (s, 2H, Ph-H), 7.53 (d, *J* = 8.2 Hz, 2H), 7.41 (d, *J* = 5.3 Hz, 1H, C7-thienopyrimidine-H), 7.01 (d, *J* = 8.5 Hz, 2H), 2.15 (s, 6H, CH_3_×2). ESI-MS: m/z 388.25 [M + H]^+^. C_21_H_17_N_5_OS (387.12)

General Synthesis Procedure for Final compounds **5a–5p**.

The amino acid, HATU and DIEA were dissolved in DMF (7 mL) and stirred at 0 °C for 15 min, followed by the addition of intermediate 4 and stirring at room temperature overnight. Then, 35 mL water was added, the mixture was extracted with EA (3 × 20 mL), and the organic phase was washed with saturated sodium chloride (3 × 30 mL). The resulting substance was dried over anhydrous Na_2_SO_4_ to give the corresponding crude product, which was purified by flash column chromatography and recrystallized from ethyl acetate (EA) and petroleum ether (PE) to give the target compounds **5a–5p**. The ^1^H NMR and ^13^C NMR of target compounds **5a–5p** were provided in Supplementary Materials.

### 3.2. In Vitro Anti-HIV Assay

The antiviral potential and cytotoxic effects of the target compounds were assessed using the MTT assay in MT-4 cells, following established protocols [20,21]. The HIV-1 (IIIB) strain, single mutant strains (L100I, K103N, Y181C, Y188L and E138K), double mutant strains (F227L + V106A and K103N + Y181C) and MT-4 cells were provided by the Rega Institute of Medicine at the University of Leuven, Belgium. These viral strains were propagated in MT-4 cells. The MT-4 cells were cultured in RPMI-1640 medium supplemented with 20 mM HEPES buffer (Life Technologies, Grand Island, NY, USA), 10% (*v*/*v*) heat-inactivated fetal calf serum (FCS), 2 mM L-glutamine, 0.1% sodium bicarbonate, and 20 μg/mL gentamicin. Cultures were maintained at 37 °C in a 5% CO_2_ atmosphere. The specific experimental procedures are detailed below.

Initially, concentrated solutions of the test compounds were prepared at 10-fold the final desired concentration and added to triplicate wells in 25 µL aliquots to assess their impacts on both mock and HIV-infected MT-4 cells. Using a Biomek 3000 robot, these were diluted in a 96-well flat-bottomed microplate by mixing with medium, creating a series of 5-fold steps down in concentration. Each test included control groups of untreated infected and mock-infected cells to establish baselines for comparison. We introduced HIV-1 strains, both wild-type (IIIB) and drug-resistant variants harboring mutations L100I, K103N, Y181C, Y188L, E138K and F227L + V106A and the double mutant K103N + Y181C (RES056), at concentrations of 100–300 CCID_50_, into their respective wells. These strains served to mimic both typical and resistant infections. Additionally, mock-infected wells received an equal volume of culture medium to assess the compounds’ impacts on uninfected cells, thereby determining cytotoxicity. MT-4 cells in the exponential growth phase were pelleted by centrifugation at 1000 rpm for 5 min, after which the supernatant was removed. The cells were resuspended to a density of 6 × 10^5^ cells/mL, and 50 µL of this suspension was added to each well. Post-infection, on the fifth day, cell viability in both mock and infected conditions was measured via the MTT assay using a spectrophotometer. The CC_50_ was identified as the concentration of the compound that caused a 50% reduction in the viability of the mock-infected cells, indicating cytotoxicity. Conversely, the EC_50_ represented the concentration that provided 50% protection against viral cytopathic effects in infected cells, signifying antiviral potency.

### 3.3. HIV-1 RT Inhibition Assay

An assay kit for HIV-1 reverse transcriptase (RT) from Roche was utilized to assess RT inhibition [22]. The kit provided all necessary components for the RT reaction, and the enzyme-linked immunosorbent assay (ELISA) methodology for the inhibition assay was executed according to the kit’s instructions. In summary, a mixture consisting of a template/primer, viral nucleotides (dNTPs) and the HIV-1 RT enzyme in an incubation buffer, with or without the test inhibitors, was incubated for 1 h at 37 °C. This was followed by transferring the mixture to a streptavidin-coated microtiter plate (MTP) for an additional hour at 37 °C to allow binding of the biotin-labeled dNTPs to streptavidin as part of the retranscription process. Unbound dNTPs were washed away, and an anti-digoxin (DIG)-POD working solution was introduced. After a 1 h incubation at 37 °C, any DIG-labeled dNTPs incorporated into the cDNA were linked to the anti-DIG-POD antibody. Excess anti-DIG-POD was removed, and a peroxidase substrate (ABTS) solution was applied to the MTPs. A color change occurred due to substrate cleavage by POD, and the absorbance was measured at 405 nm using a microplate ELISA reader. The percentage of the inhibitory activity of the RT inhibitors was calculated by formula, as given below:

% Inhibition = [O.D. value with RT but without inhibitors − O.D. value with RT and inhibitors]/[O.D. value with RT and inhibitors − O.D. value without RT and inhibitors].

The IC_50_ values corresponded to the concentrations of the test compounds required to inhibit the incorporation of biotin-dUTP into RT by 50%.

### 3.4. Molecular Simulation

Molecular simulations were executed with the Sybyl-X 2.0 software from Tripos (Certara Company, Princeton, NJ, USA). The compound **5k** was constructed employing standard geometric parameters within Sybyl-X’s Base Builder and refined using the Tripos force field, iterating over 1000 generations multiple times until the energy gradient threshold reached 0.005 kcal/(mol*Å). Gasteiger–Hückel charging was applied to the molecule. We obtained three crystallographic structures of reverse transcriptase (RT) complexes from the Protein Data Bank (PDB), including HIV-1 wild-type RT (PDB ID: 6C0J), K103N RT (PDB ID: 6C0K) and E138K RT (PDB ID: 6C0L), and prepared them for docking using Sybyl-X’s surflex-docking module. The protein structures were cleaned of ligands, water molecules and other irrelevant small molecules, and then polar hydrogens and charges were introduced. A protomol, representing the computational model of the binding site, was established, and the refined compound **5k** was docked into the NNRTI binding pocket using default parameters. Docking scores, indicative of binding affinities, were ascertained by assessing hydrophobic, polar, steric and entropic factors along with solvation effects. The top-scoring pose was rendered by the PyMOL version 1.5 software (http://www.pymol.org/) (accessed on 7 April 2022). The secondary structure of RT was shown in cartoons, and only the key residues for interactions with the inhibitor were labeled and shown in sticks. The potential hydrogen bonds were represented by dashed lines.

## 4. Conclusions

Herein, guided by the available crystallographic studies, we have designed and synthesized a novel series of amino acids (analogues)-substituted thiophene[3,2-*d*]pyrimidine derivatives by targeting the solvent-exposed region of NNIBP, with the aim of improving drug resistance profiles. Encouragingly, the anti-HIV-1 assay results demonstrated that **5k** exhibited the most potent activity against the HIV-1 WT strain and various NNRTIs-resistant strains (EC_50_ = 0.031–2.355 μM). Additionally, **5k** was endowed with lower cytotoxicity and higher SI values. The HIV-1 RT inhibition assay results demonstrated that these novel derivatives have high binding affinity for HIV-1 RT and belong to classical NNRTIs. Furthermore, preliminary SARs and molecular docking studies were also discussed in detail to provide valuable clues for further structural optimizations. Overall, all the results suggested that compound **5k** has the potential to be a lead compound for anti-HIV-1 drug development.

## Figures and Tables

**Table 1:**
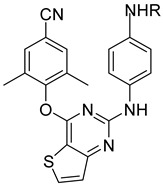
Antiviral potency against HIV-1 IIIB and RES056, cytotoxicity, and SI values of target compounds **5a**–**5p**.

Compds	R	^a^ EC_50_ (μM)		^b^ CC_50_ (μM)	^c^ SI	
		III_B_	RES056		III_B_	RES056
**5a**	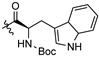	0.940 ± 0.278	>185.66	>185.66	>197	-
**5b**	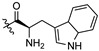	0.089 ± 0.043	>22.401	22.394 ± 0.560	250	<1
**5c**	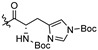	0.672 ± 0.648	>19.607	19.601 ± 0.720	29	<1
**5d**	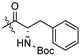	0.771 ± 0.236	>197.086	>197.086	>256	-
**5e**	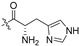	0.052 ± 0.022	≥4.884	25.105 ± 2.301	484	≤5
**5f**	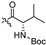	0.848 ± 0.247	>213.223	>213.223	>251	-
**5g**	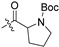	0.924 ± 0.242	>190.630	190.630	206	<1
**5h**	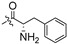	0.055 ± 0.012	8.474 ± 0.483	23.709 ± 0.521	429	3
**5i**	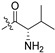	0.042 ± 0.008	≥6.623	6.535 ± 1.343	157	≤1
**5j**	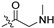	0.242 ± 0.065	>17.272	17.280 ± 11.530	72	<1
**5k**	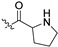	0.054 ± 0.018	7.530 ± 0.280	27.901 ± 0.910	513	4
**5l**	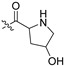	0.059 ± 0.010	>26.765	26.765 ± 1.071	451	<1
**5m**	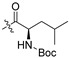	9.865 ± 1.182	>208.247	>208.247	>21	-
**5n**	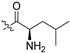	0.062 ± 0.019	5.502 ± 1.358	24.330 ± 4.280	391	4
**5o**	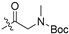	0.863 ± 0.253	-	5.844 ± 1.180	7	-
**5p**	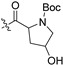	2.585 ± 0.520	-	22.532 ± 0.391	9	-
NVP		0.236 ± 0.114	>15.031	>15.031	>64	-
ETR		0.003 ± 0.001	0.040 ± 0.011	>4.608	>1387	>116
EFV		0.006 ± 0.002	0.698 ± 0.449	>6.349	>1053	>9

^a^ EC_50_: concentration of compound required to achieve 50% protection of MT-4 cell cultures against HIV-1-induced cytopathicity, as determined by the MTT method. ^b^ CC_50_: concentration required to reduce the viability of mock-infected cell cultures by 50%, as determined by the MTT method. ^c^ SI: selectivity index, the ratio of CC_50_/EC_50_.

**Table 2:** Antiviral potency against mutant HIV-1 strains of target compounds **5e**, **5h**, **5i**, **5k**, and **5l**.

Compds	^a^ EC_50_ (μM)					
	L100I	K103N	Y181C	Y188L	E138K	F227L/V106A
**5e**	0.902 ± 0.311	0.035 ± 0.009	2.585 ± 0.477	2.258 ± 0.733	0.260 ± 0.075	1.465 ± 0.979
**5h**	1.833 ± 0.208	0.073 ± 0.016	2.724 ± 0.422	2.391 ± 0.352	0.679 ± 0.569	2.113 ± 0.293
**5i**	1.199 ± 0.218	0.045 ± 0.010	>6.541	6.907 ± 0.944	0.247 ± 0.107	3.032 ± 1.755
**5k**	0.601 ± 0.261	0.031 ± 0.004	1.847 ± 0.621	2.355 ± 0.057	0.094 ± 0.022	1.755 ± 0.686
**5l**	1.481 ± 0.545	0.072 ± 0.018	2.595 ± 0.225	2.741 ± 0.103	0.314 ± 0.095	2.192 ± 0.045
**5n**	2.124 ± 0.455	0.131 ± 0.004	2.157 ± 0.057	2.111 ± 0.288	0.231 ± 0.154	2.031 ± 1.184
NVP	1.741 ± 0.879	4.091 ± 3.708	8.035 ± 1.675	9.868 ± 2.474	0.181 ± 0.067	7.499 ± 2.590
ETR	0.006 ± 0.8002	0.003 ± 0.001	0.015 ± 0.005	0.021 ± 0.008	0.009 ± 0.004	0.009 ± 0.004
EFV	0.054 ± 0.036	0.132 ± 0.050	0.008 ± 0.001	0.280 ± 0.091	0.010 ± 0.005	0.329 ± 0.228

^a^ EC_50_: concentration of compound required to achieve 50% protection of MT-4 cell cultures against HIV-1-induced cytopathicity as determined by the MTT method.

**Table 3:** Inhibitory activity against WT HIV-1 RT of target compounds **5e**, **5h**, **5i**, **5k**, **5l** and **5n**.

Compds	IC_50_ (μM)	Compds	^a^ IC_50_ (μM)
**5e**	0.927 ± 0.064	5n	2.390 ± 0.086
**5h**	1.535 ± 0.119	NVP	0.735 ± 0.152
**5i**	1.351 ± 0.148	EFV	0.012 ± 0.002
**5k**	1.124 ± 0.074	ETR	0.018 ± 0.002
**5l**	1.708 ± 0.298		

^a^ IC_50_: inhibitory concentration required to inhibit biotin deoxyuridine triphosphate incorporation into WT HIV-1 RT by 50%.

## Data Availability

The data presented in this study are available on request from the corresponding author.

## Supplementary Materials

The supporting information can be downloaded at: https://www.mdpi.com/article/10.3390/ijms25169028/s1.
