# Supplementary material for: Discovery of Novel Amino Acids (Analogues)-Substituted Thiophene[3,2-d]pyrimidine Derivatives as Potent HIV-1 Non-Nucleoside Reverse Transcriptase Inhibitors: Design, Synthesis, and Biological Evaluation
Source: Int J Mol Sci. 2024 Aug 20;25(16):9028. doi: 10.3390/ijms25169028 (PMC11354745; doi:10.3390/ijms25169028)
Supplement: Supplementary file 1 [file ijms-25-09028-s001.zip › ijms-3124595-supplementary.pdf]

Supplementary Materials

Discovery of Novel Amino Acids (Analogues)-Substituted Thiophene[3,2-*d*]pyrimidine Derivatives as Potent HIV-1 Non-Nucleoside Reverse Transcriptase Inhibitors: Design, Synthesis, and Biological Evaluation

Zongji Zhuo [1], Zhao Wang [1], Lanlan Jing [1], Tao Zhang [1], Anchao Ge [1], Zhenzhen Zhou [1], Ying Liu [1], Xin Li [1], Erik De Clercq [2], Christophe Pannecouque [2], Peng Zhan [1,3], Xinyong Liu[1,3,*] and Dongwei Kang [1,3,*]

[1]Key Laboratory of Chemical Biology (Ministry of Education), Department of Medicinal Chemistry, School of Pharmaceutical Sciences, Cheeloo College of Medicine, Shandong University, 44 West Culture Road, Jinan 250012, China

[2]Laboratory of Virology and Chemotherapy, Rega Institute for Medical Research, K.U. Leuven, Herestraat 49 Postbus 1043 (09.A097), B-3000 Leuven, Belgium

[3]China-Belgium Collaborative Research Center for Innovative Antiviral Drugs of Shandong Province, Shandong University, 44 West Culture Road, Jinan 250012, China

*Correspondence: xinyongl@sdu.edu.cn (X.L.); kangdongwei@sdu.edu.cn (D.K.)

The ^{1}H NMR and ^{13}C NMR of the target compounds

Tert-butyl ((2*S*)-1-((4-((4-(4-cyano-2,6-dimethylphenoxy)thieno[3,2-d]pyrimidin-2-yl)amino)phenyl)amino)-3-(3a,7a-dihydro-1H-indol-3-yl)-1-oxopropan-2-yl)carbamate (**5*a***). White solid, yield: 59%, mp: 205-208°C. ^{1}H NMR (400 MHz, DMSO-*d*$_6$) δ 10.81 (s, 1H, CONH), 9.89 (s, 1H, NH), 9.45 (s, 1H, NH), 8.33 (d, *J* = 5.4 Hz, 1H, C6-thienopyrimidine-H), 7.79 (s, 2H,Ph-H), 7.65 (d, *J* = 7.9 Hz, 1H), , 7.48 – 7.29 (m, 6H), 7.16 (s, 1H), 7.06 (t, *J* = 7.5 Hz, 1H), 6.98 (t, *J* = 7.4 Hz, 1H), 6.90 (d, *J* = 8.0 Hz, 1H), 4.45 – 4.25 (m, 1H), 3.11 (dd, *J* = 14.5, 5.3 Hz, 1H), 2.98 (dd, *J* = 14.5, 9.0 Hz, 1H), 2.16 (s, 6H,CH_3×2), 1.33 (s, 9H). ^{13}C NMR (100 MHz, DMSO-*d*$_6$) δ 170.67, 165.02, 162.14, 157.73, 155.34, 153.10, 137.34, 136.12, 136.05, 132.90, 132.68, 127.35, 123.83, 123.44, 120.91, 119.69, 118.70, 118.58, 118.22, 111.32, 110.08, 108.89, 78.09, 55.69, 39.99, 28.22, 27.91, 15.85. ESI-MS: m/z 676.36 [M + H]$^+$. $C_{37}H_{37}N_7O_4S$ (675.26)

(2*S*)-2-amino-N-(4-((4-(4-cyano-2,6-dimethylphenoxy)thieno[3,2-*d*]pyrimidin-2-yl)amino)phenyl)-3-(3a,7a-dihydro-1H-indol-3-yl)propenamide (**5*b***). White solid, yield: 52%, mp: 224-227 °C. ^{1}H NMR (400 MHz, DMSO-d_6)) δ 10.89 (s, 1H, NH), 9.47 (s, 1H, NH), 8.34 (d, *J* = 5.3 Hz, 1H, C6-thienopyrimidine-H), 7.79 (s, 2H), 7.61 (d, *J* = 7.8 Hz, 1H), 7.56 – 7.22 (m, 7H), 7.20 – 7.15 (m, 1H), 7.06 (t, *J* = 7.6 Hz, 1H), 6.97 (t, *J* = 7.5 Hz, 1H), 5.33 (t, *J* = 5.1 Hz, 1H, CONH), 3.50 (s, 2H), 2.15 (s, 6H, CH_3×2). ^{13}C NMR (100 MHz, DMSO-d_6) δ 173.41, 166.67, 165.02, 162.38, 162.12, 157.74, 153.10, 137.30, 136.23, 136.03, 132.87, 132.72, 132.67, 127.49, 123.78, 123.43, 120.89, 119.55, 118.58, 118.25, 111.38, 110.64, 108.89, 106.52, 56.12, 35.83, 31.04, 26.37, 15.83. ESI-MS: m/z 574.22 [M + H]$^+$. $C_{32}H_{27}N_7O_2S$ (573.19)

Tert-butyl(S)-4-(2-((tert-butoxycarbonyl)amino)-3-((4-((4-(4-cyano-2,6-dimethylphenoxy)thieno[3,2-*d*]pyrimidin-2-yl)amino)phenyl)amino)-3-oxopropyl)-1H-imidazole-1-carboxylate (**5*c***). White solid, yield: 52%, mp:236-238 °C. ^{1}H NMR (400 MHz, DMSO-d_6) δ 9.89 (s, 1H,NH), 9.45 (s, 1H, NH), 8.33 (d, *J* = 5.3 Hz, 1H, C6-thienopyrimidine-H), 8.13 (s, 1H), 7.78 (s, 2H, Ph-H), 7.48 – 7.20 (m, 6H), 7.00 (d, *J* = 8.2 Hz, 1H), 4.33 (t, *J* = 12.7 Hz, 1H), 3.03 – 2.67 (m, 2H), 2.15 (s, 6H, CH_3×2), 1.55 (s, 9H), 1.35 (s, 9H). ^{13}C NMR (100 MHz, DMSO-d_6) δ 169.90, 164.99, 162.10, 157.69, 153.06, 146.71, 137.29, 136.60, 136.13, 132.85, 132.62, 123.39, 119.65, 118.52, 108.86, 106.49, 85.15, 78.16, 30.68, 28.14, 27.39, 15.80. ESI-MS: m/z 725.37 [M + H]$^+$. $C_{37}H_{40}N_8O_6S$ (724.28)

Tert-butyl (S)-(1-((4-((4-(4-cyano-2,6-dimethylphenoxy)thieno[3,2-*d*]pyrimidin-2-yl)amino)phenyl)amino)-1-oxo-3-phenylpropan-2-yl)carbamate (**5*d***). White solid, yield: 60%, mp: 220-222 °C. ^{1}H NMR (400 MHz, DMSO-d_6) δ 9.87 (s, 1H, NH), 9.46 (s, 1H, NH), 8.33 (d, *J* = 5.3 Hz, 1H, C6-thienopyrimidine-H), 7.78 (s, 2H, Ph-H), 7.45 – 7.17 (m, 10H), 7.06 (d, *J* = 8.3 Hz, 1H, NH), 4.44 – 4.14 (m, 1H), 2.97 (dd, *J* = 7.2, 7.2 Hz, 1H), 2.83 (dd, *J* = 11.9, 11.9 Hz, 1H), 2.15 (s, 6H, CH3×2), 1.31 (s, 9H). ^{13}C NMR (100 MHz, DMSO-d_6) δ 170.24, 164.99, 162.11, 157.70, 155.37, 153.07, 138.01, 137.28, 136.14, 132.85, 132.62, 129.26, 128.03, 126.26, 123.38, 119.60, 118.55, 108.86, 106.49, 78.05, 56.40, 37.62, 28.17, 15.80. ESI-MS: m/z 635.28 [M + H]$^+$. $C_{35}H_{34}N_6O_4S$ (634.24)

(S)-2-amino-N-(4-((4-(4-cyano-2,6-dimethylphenoxy)thieno[3,2-*d*]pyrimidin-2-yl)amino)phenyl)-3-(1H-imidazol-4-yl)propenamide (**5*e***). White solid, yield: 55%, mp: 158-160 °C. ^{1}H NMR (400 MHz, DMSO-d_6) 9.84 (s, 1H, NH), δ 9.46 (s, 1H, NH), 8.33 (d, *J* = 5.4 Hz, 1H, C6-

thienopyrimidine-H), 7.79 (s, 2H, Ph-H), 7.56 (s, 1H), 7.51 – 7.22 (m, 5H), 6.84 (s, 1H), 3.61 (dd, *J* = 8.3, 5.0 Hz, 1H), 2.95 (dd, *J* = 14.5, 4.9 Hz, 1H), 2.68 (dd, *J* = 14.5, 8.2 Hz, 1H), 2.15 (s, 6H, CH_3×2). ^{13}C NMR (100 MHz, DMSO-d_6) δ 157.70, 153.09, 136.15, 134.84, 132.87, 132.69, 123.43, 119.47, 118.59, 108.87, 55.47, 29.05, 15.83. ESI-MS: m/z 525.19 $[M + H]^+$. $C_{35}H_{34}N_6O_4S$ (524.17)

Tert-butyl (S)-(1-((4-((4-(4-cyano-2,6-dimethylphenoxy)thieno[3,2-*d*]pyrimidin-2-yl)amino)phenyl)amino)-3-methyl-1-oxobutan-2-yl)carbamate (**5*f***). White solid, yield: 48%, mp: 158-160 °C. ^1H NMR (400 MHz, DMSO-d_6) δ 9.82 (s, 1H, NH), 9.45 (s, 1H, NH), 8.33 (d, *J* = 5.3 Hz, 1H, C6-thienopyrimidine-H), 7.78 (s, 2H, Ph-H), 7.50 – 7.26 (m, 5H), 6.83 (d, *J* = 8.8 Hz, 1H, NH), 3.88 (t, *J* = 8.1 Hz, 1H, COCH), 2.15 (s, 6H, CH_3×2), 2.02 – 1.90 (m, 1H), 1.38 (s, 9H), 0.88 (d, *J* = 6.7 Hz, 6H, CH_3×2). ^{13}C NMR (100 MHz, DMSO-d_6) δ 170.17, 165.01, 157.70, 153.07, 136.09, 132.86, 132.64, 119.53, 118.63, 108.85, 78.01, 30.47, 28.22, 19.24, 15.81. ESI-MS: m/z 587.26 [M + H]+. $C_{31}H_{34}N_6O_4S$ (586.24)

Tert-butyl2-((4-((4-(4-cyano-2,6-dimethylphenoxy)thieno[3,2-*d*]pyrimidin-2-yl)amino)phenyl)carbamoyl)pyrrolidine-1-carboxylate (**5*g***). White solid, yield: 45%, mp: 236-238 °C. ^1H NMR (400 MHz, DMSO-d_6) δ 9.78 (s, 1H, NH), 9.40 (s, 1H, NH), 8.32 (d, *J* = 5.4 Hz, 1H, C6-thienopyrimidine-H), 7.76 (s, 2H, Ph-H), 7.45 – 7.30 (m, 5H), 4.26 – 4.10 (m, 1H), 3.54 – 3.39 (m, 2H), 2.15 (s, 6H, CH_3×2), 2.04 – 1.77 (m, 4H), 1.28 (s, 9H). ^{13}C NMR (100 MHz, DMSO-d_6) δ 171.03, 165.02, 162.12, 157.73, 153.24, 153.09, 137.30, 136.05, 132.87, 132.62, 123.40, 119.64, 119.37, 118.61, 108.88, 106.51, 78.65, 78.48, 60.31, 59.94, 46.78, 46.59, 31.04, 28.19, 28.00, 23.44, 15.81. ESI-MS: m/z 585.30 [M + H]+. $C_{31}H_{32}N_6O_4S$ (584.22)

(S)-2-amino-N-(4-((4-(4-cyano-2,6-dimethylphenoxy)thieno[3,2-*d*]pyrimidin-2-yl)amino)phenyl)-3-phenylpropanamide (**5*h***). White solid, yield: 63%, mp: 203-206 °C. ^1H NMR (400 MHz, DMSO-d_6) δ 9.69 (s, 1H, NH), 9.42 (s, 1H, NH), 8.33 (d, *J* = 5.3 Hz, 1H, C6-thienopyrimidine-H), 7.78 (s, 2H, Ph-H), 7.53 – 7.32 (m, 5H), 7.32 – 7.12 (m, 7H), 3.57 – 3.52 (m, 1H), 3.00 (dd, *J* = 13.4, 5.4 Hz, 1H), 2.72 (dd, *J* = 13.4, 8.0 Hz, 1H), 2.15 (s, 6H, CH_3×2). ^{13}C NMR (100 MHz, DMSO-d_6)) δ 172.89, 165.02, 162.13, 157.72, 153.10, 138.69, 137.34, 136.11, 132.87, 132.68, 132.58, 129.36, 128.16, 126.18, 123.42, 119.58, 118.58, 108.87, 106.51, 56.96, 41.08, 15.84. ESI-MS: m/z 535.29 $[M + H]^{+\cdot}$ $C_{30}H_{26}N_6O_2S$ (534.18)

(*S*)-2-amino-N-(4-((4-(4-cyano-2,6-dimethylphenoxy)thieno[3,2-*d*]pyrimidin-2-yl)amino)phenyl)-3-methylbutanamide (**5*i***). White solid, yield: 50%, mp: 184-187 °C. ^1H NMR

(400 MHz, DMSO-d_6) δ 9.67 (s, 1H, NH), 9.41 (s, 1H, NH), 8.32 (d, J = 5.3 Hz, 1H, C6-thienopyrimidine-H), 7.77 (s, 2H, Ph-H), 7.50 – 7.29 (m, 5H), 3.08 (d, J = 5.4 Hz, 1H), 2.15 (s, 6H, CH_3×2), 1.97 – 1.89 (m, 1H), 0.92 (d, J = 6.8 Hz, 3H, CH_3), 0.85 (d, J = 6.8 Hz, 3H, CH_3). ^{13}C NMR (100 MHz, DMSO-d_6) δ 173.73, 165.47, 162.56, 158.18, 153.53, 137.73, 136.44, 133.30, 133.15, 133.09, 123.84, 119.96, 119.11, 118.99, 109.31, 106.92, 61.08, 32.25, 20.09, 17.76, 16.26. ESI-MS: m/z 487.33 [M + H]$^+$. $C_{26}H_{26}N_6O_2S$ (486.18)

N-(4-((4-(4-cyano-2,6-dimethylphenoxy)thieno[3,2-*d*]pyrimidin-2-yl)amino)phenyl)-2-(methylamino)acetamide (***5j***). White solid, yield: 52%, mp: 171-173 °C. ^{1}H NMR (400 MHz, DMSO-d_6) δ 9.66 (s, 1H, NH), 9.42 (s, 1H, NH), 8.33 (d, J = 5.4 Hz, 1H, C6-thienopyrimidine-H), 7.78 (s, 2H, Ph-H), 7.52 – 7.32 (m, 5H), 3.23 (s, 1H, NH), 2.32 (s, 3H, CH_3), 2.16 (s, 6H, CH_3×2), 1.90 (s, 2H). ^{13}C NMR (100 MHz, DMSO-d_6) δ 172.30, 169.06, 165.01, 162.11, 157.71, 153.09, 137.30, 136.13, 132.86, 132.65, 132.48, 123.40, 119.51, 118.62, 118.55, 108.87, 106.50, 54.22, 35.68, 21.38, 15.81. ESI-MS: m/z 459.20 [M + H]$^+$. $C_{24}H_{22}N_6O_2S$ (458.15)

N-(4-((4-(4-cyano-2,6-dimethylphenoxy)thieno[3,2-*d*]pyrimidin-2-yl)amino)phenyl)pyrrolidine-2-carboxamide (***5k***). White solid, yield: 56%, mp: 227-230°C. ^{1}H NMR (400 MHz, DMSO-d_6) δ 10.01 (s, 1H, NH), 9.49 (s, 1H, NH), 8.34 (d, J = 5.4 Hz, 1H, C6-thienopyrimidine-H), 7.79 (s, 2H, Ph-H), 7.52 – 7.32 (m, 5H), 3.86 (t, J = 7.3 Hz, 1H, CH), 3.01 (t, J = 6.7 Hz, 1H, NH), 2.15 (s, 6H, CH_3×2), 2.05 – 1.91 (m, 1H), 1.85 – 1.69 (m, 2H), 1.55 – 1.29 (m, 2H), 0.92 – 0.79 (m, 1H). ^{13}C NMR (100 MHz, DMSO-d_6) δ 172.33, 165.00, 162.10, 157.69, 153.08, 137.28, 136.14, 132.84, 132.65, 132.30, 123.39, 119.37, 118.62, 118.54, 108.86, 106.50, 60.66, 46.69, 30.97, 30.44, 25.77, 22.08, 21.24, 15.80, 13.97. ESI-MS: m/z 485.24 [M + H]$^+$ $C_{26}H_{24}N_6O_2S$ (484.17)

N-(4-((4-(4-cyano-2,6-dimethylphenoxy)thieno[3,2-*d*]pyrimidin-2-yl)amino)phenyl)-4-hydroxypyrrolidine-2-carboxamide (***5l***). White solid, yield: 50%, 246-248 °C. ^{1}H NMR (400 MHz, DMSO-d_6) δ 9.82 (s, 1H, NH), 9.44 (s, 1H, NH), 8.33 (d, J = 5.4 Hz, 1H, C6-thienopyrimidine-H), 7.78 (s, 2H, Ph-H), 7.50 – 7.32 (m, 5H), 4.76 (s, 1H, OH), 4.22 (s, 1H), 3.86 (t, J = 8.2 Hz, 1H, COCH), 2.97 – 2.88 (m, 1H), 2.84 – 2.75 (m, 1H), 2.15 (s, 6H), 2.07 – 1.97 (m, 1H), 1.82 – 1.72 (m, 1H). ^{13}C NMR (100 MHz, DMSO-d_6) δ 172.34, 165.00, 162.10, 157.69, 153.08, 137.28, 136.13, 132.84, 132.66, 132.29, 123.40, 119.38, 118.61, 118.55, 108.86, 106.51, 71.35, 59.84, 55.05, 21.23, 15.80. ESI-MS: m/z 501.33 [M + H]$^+$. $C_{26}H_{24}N_6O_3S$ (500.16)

Tert-butyl (*R*)-(1-((4-((4-(4-cyano-2,6-dimethylphenoxy)thieno[3,2-*d*]pyrimidin-2-yl)amino)phenyl)amino)-4-methyl-1-oxopentan-2-yl)carbamate (**5*m***). White solid, yield: 60%, 146-149 °C. ^{1}H NMR (400 MHz, DMSO-d_6) δ 9.78 (s, 1H, NH), 9.44 (s, 1H, NH), 8.33 (d, *J* = 5.3 Hz, 1H, C6-thienopyrimidine-H), 7.78 (s, 2H, Ph-H), 7.50 – 7.27 (m, 5H), 6.98 (d, *J* = 8.1 Hz, 1H), 4.18 – 4.01 (m, 1H), 2.15 (s, 6H, CH_3×2), 1.71 – 1.47 (m, 2H), 1.38 (s, 9H), 1.22 (s, 1H), 0.89 (dd, *J* = 6.6, 3.7 Hz, 6H, CH_3×2). ^{13}C NMR (100 MHz, DMSO-d_6) δ 164.99, 162.08, 157.70, 153.06, 137.26, 135.96, 132.83, 132.76, 132.62, 123.37, 119.40, 118.58, 118.53, 108.84, 106.46, 53.75, 44.07, 24.22, 23.20, 21.89, 15.79. ESI-MS: m/z 601.30 [M + H]$^+$. $C_{32}H_{36}N_6O_4S$ (600.25)

(*R*)-2-amino-N-(4-((4-(4-cyano-2,6-dimethylphenoxy)thieno[3,2-*d*]pyrimidin-2-yl)amino)phenyl)-4-methylpentanamide (**5*n***). White solid, yield: 62%, 138-140°C. ^{1}H NMR (400 MHz, DMSO-*d*6) δ 9.76 (s, 1H, NH), 9.44 (s, 1H, NH), 8.33 (d, *J* = 5.3 Hz, 1H, C6-thienopyrimidine-H), 7.78 (s, 2H, Ph-H), 7.59 – 7.19 (m, 5H), 3.33 – 3.25 (m, 1H), 2.15 (s, 6H, CH_3×2), 1.80 – 1.66 (m, 1H), 1.52 – 1.24 (m, 2H, CH2), 0.89 (dd, *J* = 10.7, 6.6 Hz, 6H, CH_3×2). ^{13}C NMR (100 MHz, DMSO-$d_{6)}$ δ 164.99, 162.08, 157.70, 153.06, 137.26, 135.96, 132.83, 132.76, 132.62, 123.37, 119.40, 118.58, 118.53, 108.84, 106.46, 53.75, 44.07, 24.22, 23.20, 21.89, 15.79. ESI-MS: m/z 501.34 [M + H]$^+$. $C_{27}H_{28}N_6O_2S$ (500.20)

Tert-butyl(2-((4-((4-(4-cyano-2,6-dimethylphenoxy)thieno[3,2-*d*]pyrimidin-2-yl)amino)phenyl)amino)-2-oxoethyl)(methyl)carbamate (**5*o***). White solid, yield: 57%, 162-164 °C. ^{1}H NMR (400 MHz, DMSO-d_6) δ 9.90 – 9.71 (m, 1H, NH), 9.45 (s, 1H, NH), 8.33 (d, *J* = 5.3 Hz, 1H, C6-thienopyrimidine-H), 7.78 (s, 2H, Ph-H), 7.50 – 7.25 (m, 5H), 3.91 (d, *J* = 17.5 Hz, 2H), 2.86 (d, *J* = 10.4 Hz, 3H, CH_3), 2.15 (s, 6H, CH_3×2), 1.44 – 1.30 (m, 9H). ^{13}C NMR (100 MHz, DMSO-d_6) δ 167.21, 166.96, 165.00, 162.10, 157.71, 155.44, 155.18, 153.07, 137.26, 136.03, 132.84, 132.70, 132.60, 123.37, 119.50, 119.29, 118.63, 118.52, 108.86, 106.48, 78.83, 78.66, 52.19, 51.31, 35.73, 35.64, 30.97, 28.06, 27.97, 22.07, 15.79, 13.95. ESI-MS: m/z 559.27 [M + H]$^+$. $C_{29}H_{30}N_6O_4S$ (558.20)

Tert-butyl2-((4-((4-(4-cyano-2,6-dimethylphenoxy)thieno[3,2-*d*]pyrimidin-2-yl)amino)phenyl)carbamoyl)-4-hydroxypyrrolidine-1-carboxylate (**5*p***). White solid, yield: 53%, 233-235 °C. ^{1}H NMR (400 MHz, DMSO-d_6) δ 9.89 (s, 1H, NH), 9.45 (s, 1H, NH), 8.33 (d, *J* = 5.3 Hz, 1H, C6-thienopyrimidine-H), 7.77 (s, 2H, Ph-H), 7.47 – 7.17 (m, 5H), 5.07 (t, *J* = 3.1 Hz, 1H, COCH), 4.34 – 4.23 (m, 2H), 3.53 – 3.38 (m, 2H), 2.15 (s, 6H, CH_3×2), 1.95 – 1.85 (m, 1H), 1.40

– 1.25 (m, 9H). ^{13}C NMR (100 MHz, DMSO-d_6) δ 162.09, 157.69, 153.06, 132.85, 132.61, 119.48, 118.51, 108.85, 78.53, 39.99, 28.17, 27.95, 15.80. ESI-MS: m/z 601.37 [M + H]$^+$. $C_{31}H_{32}N_6O_5S$ (600.22)
